# ADMSC Exo‐MicroRNA‐22 improve neurological function and neuroinflammation in mice with Alzheimer's disease

**DOI:** 10.1111/jcmm.16787

**Published:** 2021-07-11

**Authors:** Liping Zhai, Heping Shen, Yongjia Sheng, Qiaobing Guan

**Affiliations:** ^1^ Department of Neurology The Second Affiliated Hospital of Jiaxing University Jiaxing China; ^2^ Department of Pharmacy The Second Affiliated Hospital of Jiaxing University Jiaxing China

**Keywords:** Alzheimer's disease, exosomes, mesenchymal stem cells, miRNA‐22, pyroptosis

## Abstract

The previous study by our group has found that miRNA‐22 can inhibit pyroptosis by targeting GSDMD and improve the memory and motor ability of mice with Alzheimer's disease (AD) mice by inhibiting inflammatory response. In recent years, stem cells and their exosomes have been reported to have good therapeutic effects on AD; therefore, we hypothesize that miRNA‐22 is likely to play a synergistic therapeutic effect. In this study, adipose‐derived mesenchymal stem cells (ADMSCs) were transfected into miRNA‐22 mimic to obtain miRNA‐22 loaded exosomes (Exo‐miRNA‐22), which was further used for the treatment and nerve repair of AD. In brief, 4‐month‐old APP/PS1 mice were assigned into the control group, Exo and Exo‐miRNA‐22 groups. After exosome transplantation, we observed changes in the motor and memory ability of mice. In addition, ELISA was used to detect the expression of inflammatory factors in cerebrospinal fluid and peripheral blood, Nissl staining was used to assess the survival of mouse nerve cells, immunofluorescence staining was used to determine the activation of microglia, and Western blot was utilized to detect the expression of pyroptosis‐related proteins. As a result, the nerve function and motor ability were significantly higher in mice in the Exo‐miRNA‐22 group than those in the control group and Exo group. Meanwhile, the survival level of nerve cells in mice was higher in the Exo‐miRNA‐22 group, and the expression of inflammatory factors was lower than that of the Exo group, indicating Exo‐miRNA‐22 could significantly suppress neuroinflammation. In vitro culture of PC12 cells, Aβ_25‐35_‐induced cell damage, detection of PC12 apoptotic level, the release of inflammatory factors and the expression of pyroptosis‐related proteins showed that Exo‐miRNA‐22 could inhibit PC12 apoptosis and significantly decrease the release of inflammatory factors. In this study, we found that miRNA‐22‐loaded ADMSC‐derived exosomes could decrease the release of inflammatory factors by inhibiting pyroptosis, thereby playing a synergetic therapeutic role with exosomes on AD, which is of great significance in AD research.

## 1 BACKGROUND

Alzheimer's disease (AD) is a neurodegenerative disease. Since the first report on AD by German doctor Alzheimer in 1907, there has been limited therapeutic progress in AD.^1^, ^2^ In recent years, with deepened research on stem cells, accumulative studies have shown that stem cells have good therapeutic effect on AD.^3^, ^4^ Stem cells have the potential for self‐renewal and differentiation. In 1998, embryonic stem cells were obtained, expanded in vitro and subsequently transplanted into the brain of AD mice to replace lost and damaged nerve cells to further repair the nervous system; and the underlying mechanism was associated with the differentiation of neuron‐like cells and the secretion of neurotrophic factor, which can promote endogenous repair, etc.^5^, ^6^ However, due to the multi‐directional differentiation function, transplantation of stem cells may cause adverse effects such as tissue immune rejection, teratogenicity and tumorigenesis. In addition, the survival rate of stem cells after transplantation is also a great concern. Therefore, it is urgently needed to explore a new alternative approach on stem cell therapy. Exosomes are a type of vesicle‐like structure secreted by cells. As an important medium for inter‐cellular communication, exosomes are involved in the regulation of biological signals by binding with target cells through membrane receptors. At present, relevant studies have also shown that stem cell‐derived exosomes have a good therapeutic effect on AD.^7^, ^8^

MicroRNAs (miRNAs) are a class of non‐coding RNA with a length of 18‐22nt. miRNAs can regulate the production of target protein by recognizing the 3’UTR sequence on the target gene mRNA to further suppress the transcription and translation of mRNA. At present, miRNAs have been reported to play important roles in various diseases. Our previous studies have found that miRNA‐22 can target GSDMD and relieve the inflammatory response of AD by inhibiting pyroptosis.^9^ Therefore, in this study, adipose‐derived mesenchymal stem cells (ADMSCs)‐derived exosomes were loaded with miRNA‐22 and can relieve the ethology of AD through multiple aspects of effects such as inhibiting inflammation and nerve repair, which was considered as a novel idea for AD treatment.

## 2 MATERIALS AND METHODS

### 2.1 miRNA‐22‐loaded mouse ADMSCs and miRNA‐22 and exosome extraction

Mouse ADMSCs (Wuhan Punosai Biotechnology Co., Ltd.) were cultured in DMEM complete medium (Gibco) at 37℃ with 5% CO2. ADMSCs were collected at the logarithmic phase, followed by transfection miRNA‐22. ADMSCs were inoculated into 6‐well plates. Opti‐MEM medium and HiPerFect transfection reagent were mixed; 250μl of Opti‐MEM and 10μl of miRNA‐22 mimic were mixed and incubated for 5 min. The transfection reagent and plasmid were mixed and incubated for 20 min. After discarding the medium, cells were cultured with the mixed Opti‐MEME. After transfection for 72h, cells were regularly cultured. For exosome extraction, miRNA‐22 mimic‐transfected ADMSCs were digested and washed with PBS for three times. DMEM complete medium was added with exosome‐free 10% FBS (Gibco, Massachusetts, USA). ADMSCs were cultured for 72 h to collect conditional medium, followed by isolation of exosomes by ultra‐high‐speed centrifugation.

### 2.2 Identification of ADMSCs exosome

Identification of exosomes isolated from the ADMSC medium: (1) Morphological observation of exosomes by electron microscopy: exosomes obtained from 40 ml culture medium by ultracentrifugation were resuspended in 20–30 μl PBS. Afterwards, 10 μl of the sample was dropped on the copper net for precipitation for 1 min. The floating liquid was absorbed by filter paper. 10 μl of 2% uranium acetate was added on the copper net for 1 min, followed by absorption of the floating liquid by the filter paper and dry at room temperature for several minutes. Finally, samples were subjected to photography at 80–120 kv. (2) Analysis of exosome particle size: the number and size of exosomes were directly tracked by the rate of Brownian motion of exosomes using the NanoSight NS 300 system (NanoSight Technology, Malvern, UK), configured with a high‐sensitivity sCMOS camera, fast video capture and particle‐tracking software (NanoSight). The samples were diluted 150–3000 times with Dulbecco's PBS (DPBS) without any nanoparticles to attain a concentration of 1–20 × 10^8^ particles per millilitre for analysis. Each sample was measured in triplicate at the camera, which recorded and tracked each visible particle. Exosome numbers and size distribution were explored using the Stokes‐Einstein equation. (3) Detection of exosome marker protein: exosomes and ADMSCs were lysed with NP‐40 (Beyotime) to extract protein, followed by protein quantification using BCA kit (Beyotime). The protein was supplemented with 5× loading buffer up to 20 μl and boiled for 8 min. Protein samples were subjected to electrophoresis at 80 V and then switched to 120 V. After removing the gel, protein was transferred to the PVDF membrane at 300 mA constant current for 0.5–2 h. The PVDF membrane was blocked with 5% skim milk powder for 2h and incubated with proper primary antibodies diluted in TBST (CD63, CD81 and ALIX). After incubation, the PVDF membrane was washed with TBST twice and incubated with HRP‐labelled goat anti‐rabbit secondary antibody (Abcam). After incubation, the chemiluminescence method was used to determine the optical density using Image Pro‐Plus 6.0 software. (4) The expression level of miRNA‐22 by quantitative PCR (qPCR): exosomes were added with 1 ml of lysis buffer, incubated at room temperature for 5 min, added with 0.2 ml of chloroform, incubated at room temperature for 5 min, and centrifuged at 2500 *g* at 4℃ for 10 min. The upper aqueous phase was transferred to a new tube and added with the same volume of 70% ethanol for RNA precipitation. The precipitated RNA was added to the adsorption column for purification, the purified RNA was added to a 1.5‐ml centrifuge tube, followed by addition of 40 μl of RNase Free water, incubation at room temperature for 2 min, centrifugation at 2500 *g* for 1 min and store in the −80℃ refrigerator. RevertAid First Strand cDNA Synthesis Kit (Fermentas MBI, USA) was used for reverse transcription of cDNA. The reaction conditions were as follows: 37℃, 15 min; 85℃, 5 s; 4℃ for storage. The primer sequences of miRNA‐22 were as follows: F: 5′‐AAGCTGCCAGTTGAAGAACTGTA‐3′; R: 5′‐GCTGTCAACGATACGCTACGTAAC‐3′. The primer sequences of U6 (internal control) were as follows, F: 5′‐CGCTTCGGCAGCACATATA‐3′; R: 5′‐TTCACGAATTTGCGTGTCAT‐3′. The relative expression of miRNA‐22 was calculated using the 2^−∆∆Ct^ method, ∆Ct = −Ct(miRNA‐22)‐Ct(U6), ∆∆Ct = (Ct of target gene‐Ct of internal control) experimental group‐(Ct of target gene‐ Ct internal control) control group.

### 2.3 The intervention method and the detection of behaviour and neuroinflammation in AD mice

APP/PS1 double transgenic mice (Jackson, USA) were kept in Animal Experiment Center of JiaXing University. A total of 30 4‐month‐old APP/PS1 double transgenic mice were assigned into the Control, Exo and Exo‐miRNA‐22 groups. To be specific, APP/PS1 mice in the Control group were routinely maintained and injected with normal saline as control. Exosomes used in the Exo group were ADMSC‐derived exosomes without transfection with miRNA‐22 mimic. Exosomes used in the Exo‐miRNA‐22 group were miRNA‐22‐loaded exosomes after miRNA‐22 mimic transfection. Exo and Exo‐miRNA‐22 were injected into the tail vein every 7 days at a dose of 100 μg/ml (50 μl each time), whereas mice in the Control group were injected with 50 μl normal saline each time.

Morris water maze test was used to test the memory ability of mice. Morris water maze and video system (Feidi Biotechnology Co., Ltd.) were used. In brief, the water maze consisted of a circular pool, platform and recording system. The pool had a diameter of 120 cm, a height of 40 cm and a water depth of 30 cm. The inner wall of the pool was black, and the temperature was maintained at 20℃. The experiment was performed 1 cm underwater and the experimental platform was at the mid‐point of the four quadrants. Mice were subjected to adaptive training one day before the experiment. Each mouse was trained for one time, which was allowed to swim from the entrance for 60 s. If the mouse could not find the platform, it was artificially guided to stand on the platform for 20 s. If the mouse was able to find the platform, then, after standing on the platform for 20 s, it was placed back cage. For navigation test, which lasted for four weeks (30 days), each mouse was examined four times a day (two times in the morning and another two times in the afternoon). The platform was placed in the fourth quadrant. The time interval of entrance with finding and boarding the platform was recorded. If the mouse could not find the platform within 60 s, then it was guided to the platform and further stood for 20 s. Escape latency (EL) was defined as the time interval between entrance into the water and finding the platform. In terms of space exploration experiment, the platform was removed after the navigation test, and mouse was allowed to enter the entrance. Afterwards, investigators recorded the times of crossing the fourth quadrant and the time of staying on the original platform within 60 seconds. The behavioural test of mice was carried out 5–10–15–20–25–30 days after exosome transplantation.

After 30 days of behavioural detection, mice were sacrificed by carbon dioxide, followed by extraction of the brain tissue, peripheral blood and cerebrospinal fluid (CSF) of mice.

1. Enzyme‐linked immunosorbent assay (ELISA) was used to detect the expression of inflammatory factors, including IL‐1β, IL‐6 and TNF‐α in peripheral blood and CSF: CSF and peripheral blood were centrifuged to collect supernatant for storage. IL‐1β, IL‐6 and TNF‐α were detected by ELISA kit (Nanjing Jiancheng Biotechnology Co., Ltd.) according to the manufacturers’ instruction. A microplate reader (BioTek, USA) was used to detect the absorbance at 450nm, and the result was shown as pg/ml.
2. Nissl staining was used to detect the distribution of nerve cells: Paraffin‐embedded mouse brain tissue was cut into slices, dewaxed with xylene for three times (10 min each), hydrated with absolute ethanol, 90% ethanol and 70% ethanol for 2 min each and washed with distilled water for 2 min. Tissues were stained with Nissl staining solution (dropwise) for 5 min, washed twice with distilled water, dehydrated with 95% ethanol, treated with xylene for 5 min and finally observed under microscope after sealing with neutral resin.
3. Immunofluorescence (IF) staining of microglia IBA‐1 and CD206: After sacrificing mice using carbon dioxide suffocation, the brain tissue was fixed with 4% paraformaldehyde after perfusion fixation and subsequently dehydrated with 15% and 30% sucrose solution. After OCT embedding, the tissue section was continuously cut into 8‐μm‐thick slices, which were stored at −20℃ for further use. The sections were washed with PBS, blocked with 5% serum for 30 min, incubated with CD206 (1:50) and IBA‐1 (1:50) monoclonal antibodies (Abcam) at 4℃ overnight, washed with PBS for three times, incubated with fluorescent antibody (1:50) for 1h in dark, washed with PBS for three times, mounted with anti‐fluorescence quencher and observed under a microscope. CD206 showed red fluorescence, and IBA‐1 showed green fluorescence. Image Pro‐Plus was used to count the number of positive cells.
4. Western blot analysis was used to detect the relative expression of protein: After grinding the tissue with liquid nitrogen, 1.0ml pre‐chilled RIPA lysate (Beyotime Biotechnology Co., Ltd) was added to lyse tissue on ice for 30min, followed by centrifugation at 10,000 g for 15 min. The supernatant was subjected to protein quantification by BCA kit (Beyotime Biotechnology Co., Ltd). The protein was supplemented with 5x loading buffer up to 20 μl and boiled for 8min. Protein samples were subjected to electrophoresis at 80V and then switched to 120 V. After removing the gel, protein was transferred to the PVDF membrane at 300 mA constant current for 0.5–2 h. The PVDF membrane was blocked with 5% skim milk powder for 2 h and incubated with proper primary antibodies diluted in TBST. After incubation, the PVDF membrane was washed with TBST twice and incubated with HRP‐labelled goat anti‐rabbit secondary antibody (Abcam). After incubation, the chemiluminescence method was used to determine the optical density using Image Pro‐Plus 6.0 software. GAPDH was used as the internal control, and the results were shown as the comparison of the optical density values between the target protein and the internal control protein. The primary antibodies were as follows: monoclonal antibodies against key proteins of pyroptosis, GSDMD, p30‐GSDMD (1:500) (Abcam), monoclonal antibodies against key protein of inflammasome, NLRP3, Caspase‐1 and pro‐Caspase‐1 (1:800) (Abcam). The dilution concentration of HRP‐labelled IgG antibody is 1:1000 (Abcam).

### 2.4 Aβ25‐35‐induced PC12 cell injury and intervention

PC12 cells (mice cortical neurons, MCN) (Procell) were cultured with RPMI‐1640 (Procell, Wuhan, China). When cells grew to about 70%, cells were divided into Control group, Aβ_25‐35_ group, Exo group and Exo‐miRNA‐22 group. Cells in the Aβ_1‐42_ group were treated with 25μM Aβ_1‐42_ (Sigma, USA). PC12 cells were regularly maintained in the Control group. Cells in the Exo group and Exo‐miRNA‐22 group were pretreated with exosomes at a final concentration of 15 μg/mL (protein amount) for 12 h, followed by intervention with Aβ_1‐42_ (25 μM).

### 2.5 Intervention effect of Exosome‐miRNA‐22 on PC12 cell injury

1. Cytotoxicity detection by lactate dehydrogenase (LDH) method: LDH kit (Solarbio) was used to detect cytotoxicity. Briefly, LDH release rate was determined on MCN after 12‐h intervention with Aβ_1‐42_ (shown as %).
2. Pyroptosis level by propidium iodide (PI) and Hoechst 33,258 staining: PC12 cells were stained after Aβ intervention for 12 h. After discarding medium, cells were washed with PBS for two times, incubated with Hoechst 33,258 staining solution (Beyotime Biotechnology Co., Ltd.) (dilution 1:100) for 15 min, washed with PBS for two times, followed by observation under microscopy (positive cells showed blue fluorescence). For PI staining, cells were incubated with PI staining reagent (Beyotime Biotechnology Co., Ltd., Shanghai, China) at 1 μg/ml for 30 min and washed with PBS for two times (positive cells showed red fluorescent). The above two staining methods were used to detect the number of pyroptotic cells.
3. Immunofluorescence (IF) staining for GSDMD: IF staining was performed using cell slide method. In brief, the coverslip was placed in a 6‐well plate and MCN cells were inoculated. When cells grew into 70% confluency, pyroptosis was induced. After incubation for 2 h, cells were fixed with freshly prepared 4% paraformaldehyde for 10 min, washed with PBS for three times, permeabilized with 0.2% Triton X‐100 for 10 min, blocked with 2% BSA for 30 min, incubated with GSDMD monoclonal antibody (dilution 1:300; Abcam) at room temperature for 1 h, washed with PBS for three times and incubated with IgG antibodies (Abcam). Finally, 0.5μg/ml DAPI staining reagent (Solarbio, Beijing, China) was used for nuclear staining. After washing with PBS for two times, the slide was mounted and observed under a fluorescent microscope.
4. Detection of relative protein expression by Western blot analysis: After intervention with Aβ_1‐42_ for 12 h, PC12 cells were collected, washed with PBS for two times and lysed with pre‐chilled 1.0 ml RIPA lysate (Beyotime Biotechnology Co., Ltd) on ice for 30 min, followed by centrifugation at 10,000g for 15 min. The detection method was the same as that of tissue proteins.
5. Expression of inflammatory factors in the culture medium: ELISA was used to detect the expression of inflammatory factors in the culture medium. Briefly, PC12 cells were collected after Aβ_1‐42_ intervention for 12 h, followed by centrifugation at 3000g to collect supernatant to detect inflammatory factors. IL‐1β, IL‐18 and TNF‐α were tested by ELISA kit (Nanjing Jiancheng Biotechnology Co., Ltd) according to the manufacturer's instruction. The absorbance was determined at a wavelength of 450 nm using a microplate reader (BioTek) (shown as pg/ml).
6. Detection of apoptotic level by Annexin‐FITC/PI flow cytometry: Cells were cultured in 6‐well plates. When cells reached to 60%–70% confluency, the old medium was discarded, followed by incubation with fresh medium. After Aβ_1‐42_ treatment for 12 h, cells were collected and resuspended in PBS, followed by counting. Resuspended cells were centrifuged at 200g for 5min; the supernatant was discarded, followed by addition of 195 μl Annexin V‐FITC binding solution and 5μl Annexin V‐FITC. After incubation at room temperature in dark for 10 min, cells were subjected to flow cytometry. Annexin V‐FITC showed green fluorescence and PI showed red fluorescence.

### 2.6 Statistical analysis

All measurement data were shown as $\bar{x}$±s. SPSS 17.0 was used for statistical analysis. After homogeneity of variance test, two independent samples t test was used for comparison between two groups. One‐way ANOVA was used for comparison among three groups or more, followed by LSD method for comparison between two groups. A two‐sided *p* <.05 indicated statistical significance.

## 3 RESULTS

### 3.1 Characterization of exosomes and identification of miRNA‐22 load

**FIGURE 1:**
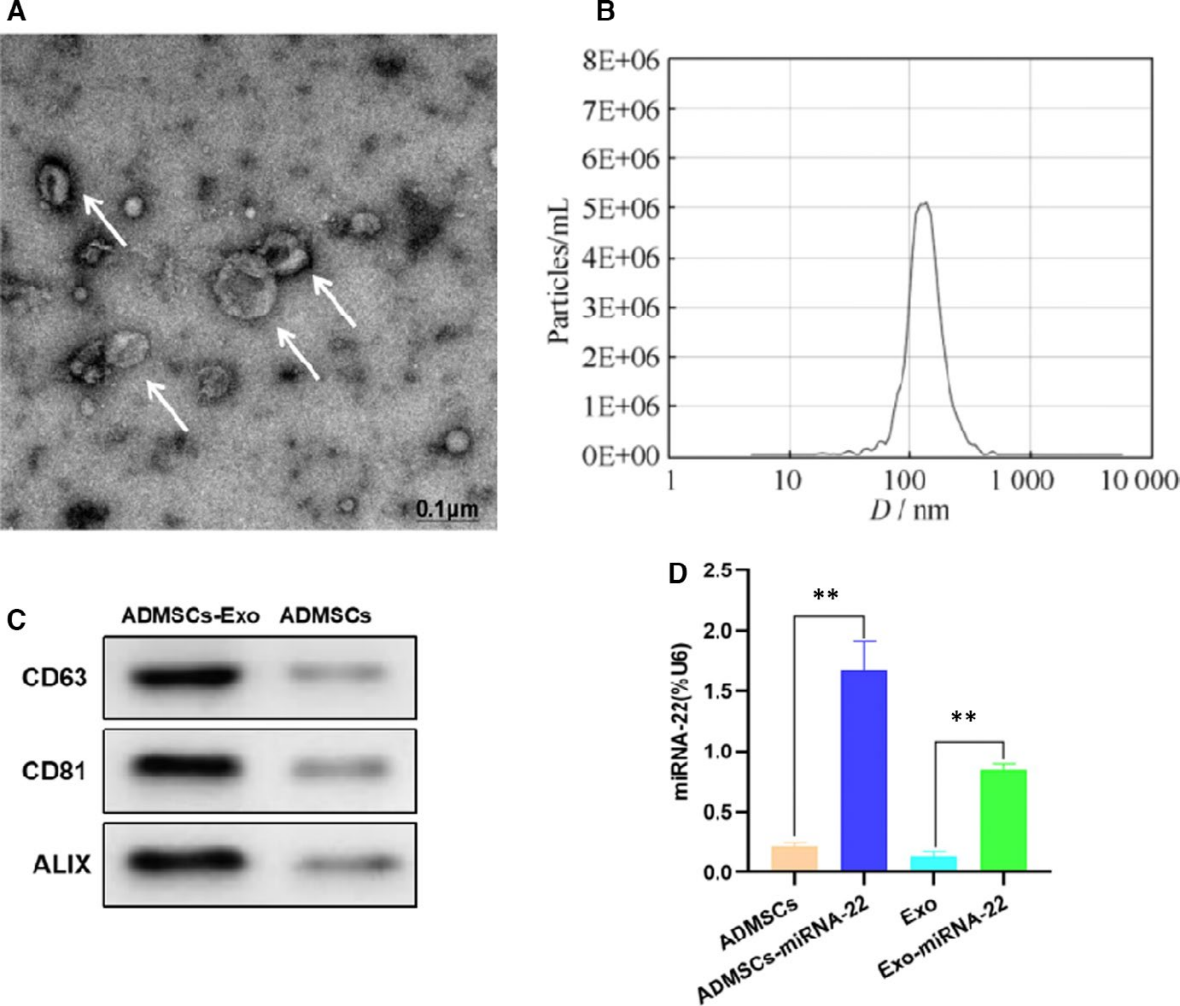
Exosome characterization and miRNA‐22 expression. A: Exosome morphology (n = 3): Exosome morphology was pie‐like and vesicle‐like. B: Exosome particle size analysis (n = 3): The particle size exosomes were distributed in (112.76 ± 15.44) nm. C: Exosome marker protein (n = 3): The expression of CD63, CD81 and ALIX in ADMSCs Exo was significantly higher than that of ADMSCs. D: Expression of miRNA‐22 ($\bar{x}$±s, n = 3): After transfection of mimics into ADMSCs‐miRNA‐22, the expression of miRNA‐22 was significantly higher than that of ADMSCs, whereas the expression of miRNA‐22 in Exo‐miRNA‐22 was higher than Exo. Comparison between groups, **p <.01

The morphology of isolated exosomes was in pie‐like, vesicle‐like structure, and particle size analysis showed that the size distribution of exosomes was 112.76 ± 15.44 nm. In addition, the expression of exosome marker proteins CD63, CD81 and ALIX was high in exosomes, which was significantly higher compared with ADMSCs. Therefore, the extracted exosomes conformed to the morphological characteristics (Figure 1A–C), which can be determined as exosomes for subsequent experiments. The detection results of miRNA‐22 showed that after mimics transfection into ADMSCs, the expression of miRNA‐22 in cells was significantly up‐regulated, which was significantly higher than that of ADMSCs (*p* <.05). The expression of miRNA‐22 in Exo‐miRNA‐22 was significantly higher than that in Exo, indicating that the exosomes secreted by ADMSCs‐miRNA‐22 were successfully loaded with miRNA‐22 (Figure 1D).

### 3.2 Behavioural changes, inflammatory factor expression and Nissl staining in APP/PS1 mouse

**FIGURE 2:**
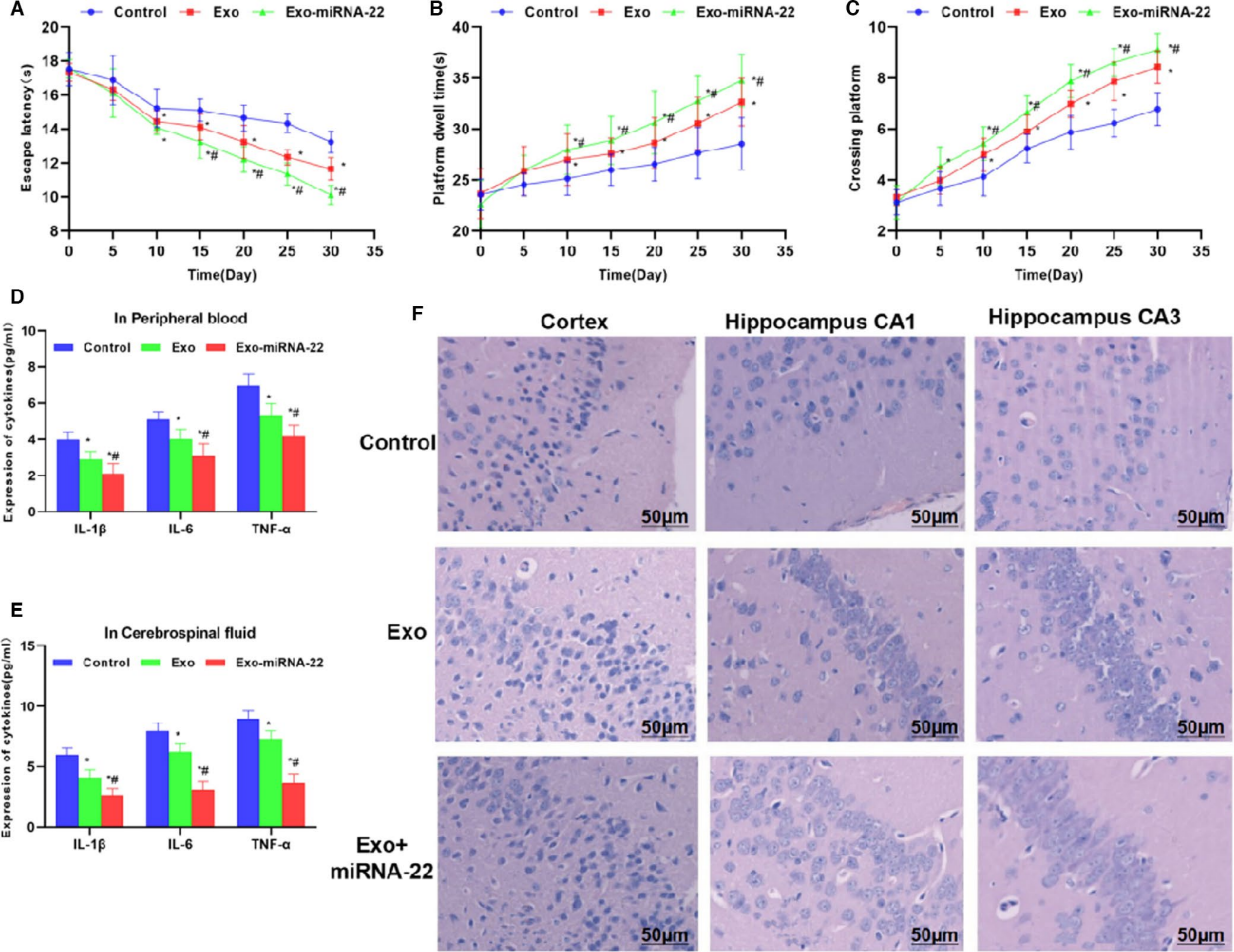
Behavioural changes, inflammatory factor expression and Nissl staining in mice. A: Escape time in water maze assay ($\bar{x}$±s, n = 10): With the extension of time, the escape time was significantly shortened in mice of the three groups. Comparison with the Control group, *p <.05; comparison with the Exo group, ^#^ *p* <.05. B: Platform staying time in water maze assay ($\bar{x}$±s, n = 10): With the extension of time, the platform staying time was significantly prolonged in mice of the three groups. Comparison with the Control group, *p <.05; comparison with the Exo group, ^#^ *p* <.05. C: Platform crossing times in water maze assay ($\bar{x}$±s, n = 10): With the extension of time, the platform crossing times were significantly increased in mice of the three groups. Comparison with the Control group, *p <.05; comparison with the Exo group, ^#^ *p* <.05. D‐E: Detection of inflammatory factors expression in the peripheral blood and cerebrospinal fluid of mice ($\bar{x}$±s, n = 10): The expression of inflammatory factors was relatively high in the Control group, whereas the expression of inflammatory factors was down‐regulated in the Exo and Exo‐miRNA‐22 groups. Moreover, the expression was further down‐regulated in the Exo‐miRNA‐22 group, compared with Control group, comparison with the Control group, *p <.05; comparison with the Exo group, ^#^ *p* <.05. F: Nissl staining (n = 3): Nerve cells were sparsely arranged in the cortex and hippocampus CA1 and CA3 of the Control group, with slight staining and obvious nerve damage. Nerve cells were densely arranged in the Exo and Exo‐miRNA‐22 groups than the Control group, with deep staining and significant alteration

In the behavioural changes of mice, there was significant cognitive impairment in mice of the Control group. With the repeated conduction of water maze assay, the behavioural and memory abilities of mice were improved to a certain extent. In the detection of escape time, platform staying time and platform crossing times, with the extension of time, the escape time was shortened, the platform staying time and platform crossing times were increased in mice of the Exo group, indicating the improved behavioural and memory abilities, which was significantly different from the Control group (*p* <.05). The escape time was further shortened, and the platform staying time and platform crossing times were further increased in mice of the Exo‐miRNA‐22 group, which were significantly different from the Exo group (*p* <.05) (Figure 2A‐C). The detection of inflammatory factors showed that the expression levels of IL‐1β, IL‐6 and TNF‐α in the peripheral blood and cerebrospinal fluid were relatively high in the Control group. After Exo intervention, the expression of inflammatory factors was significantly down‐regulated compared with the Control group (*p* <.05). Moreover, Exo‐miRNA‐22 intervention could further decrease the expression of inflammatory factors; especially, the inflammatory factors in the cerebrospinal fluid were significantly down‐regulated, which is significantly different from the Exo group (*p* <.05) (Figure 2D‐E). Nissl staining was used to detect nerve cell injury. As a result, nerve cells were sparsely arranged in the cortex and hippocampus CA1 and CA3 in mice of the Control group, indicating obvious damage. Cells in the Exo and Exo‐miRNA‐22 groups were densely arranged and positively stained compared with the Control group, which was significantly improved compared with the Control group, indicating that Exo and Exo‐miRNA‐22 treatment can further inhibit nerve cell damage (Figure 2F).

### 3.3 Microglia activation and protein expression in mice

**FIGURE 3:**
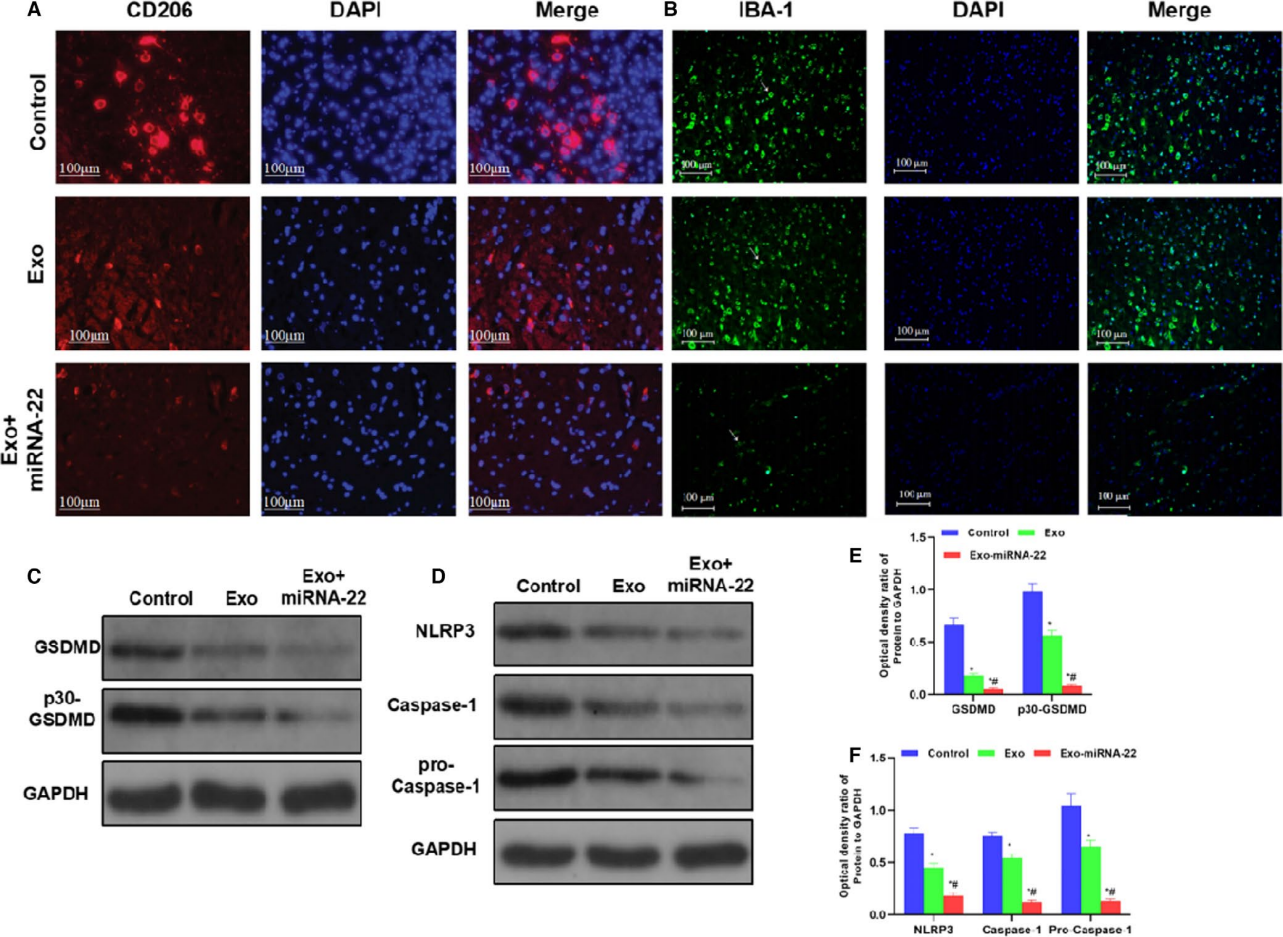
Detection of microglia activation and protein expression in mice. A‐B: Immunofluorescence staining of CD206 and IBA‐1 in mice (n = 3): The expression of CD206 and IBA‐1 was relatively high in the Control group, with relatively high fluorescence intensity. The expression was down‐regulated in the Exo group, with decreased fluorescence intensity of CD206 and IBA‐1, which was significantly lower than that in the Control group. The fluorescence intensity of CD206 and IBA‐1 was lower in the Exo‐miRNA‐22 group than that in the Control group and Exo group. C‐F: Detection of protein expression ($\bar{x}$±s, n = 3): The expression of GSDMD and p30‐GSDMD was relatively high in the Control group, which was significantly down‐regulated in the Exo and Exo‐miRNA‐22 groups. Meanwhile, the expression of NLRP3 inflammasome key proteins (NLRP3 and Caspase‐1) was relatively high in the Control group, which was significantly down‐regulated in the Exo and Exo‐miRNA‐22 groups. Comparison with the Control group, *P <.05; comparison with the Exo group, ^#^ *P* <.05

The expression levels of CD206 and IBA‐1 were relatively high in the Control group, indicating the obvious activation of microglia. The high expression of inflammatory factors was closely associated with microglia activation, and the excessive activation of microglia was the main cause for the release of inflammatory factors. The expression of CD206 and IBA‐1 was significantly down‐regulated in the Exo group compared with that in the Control group. The expression of CD206 and IBA‐1 was significantly down‐regulated in the Exo‐miRNA‐22 group compared with that in the Exo and Control groups (Figure 3A‐B). The protein detection results showed that the GSDMD protein was activated in the Control group, the expression of p30‐GSDMD was relatively high, and NLRP3 inflammasome was also activated. The expression of NLRP3 and Caspase‐1 was up‐regulated, which was positively correlated with GSDMD cleavage. Meanwhile, the expression of GSDMD and p30‐GSDMD was suppressed, and the protein expression of NLRP3 was down‐regulated in the Exo group, which was significantly different from that in the Control group. In the Exo‐miRNA‐22 group, due to the targeted intervention on GSDMD by miRNA‐22, the expression of GSDMD and p30‐GSDMD was relatively low, the expression of NLRP3 was also significantly down‐regulated, which was significantly different compared with the Exo and Control groups (*p* <.05) (Figure 3C‐F).

### 3.4 Intervention effect of miRNA‐22‐loaded exosomes on PC12 cell injury

**FIGURE 4:**
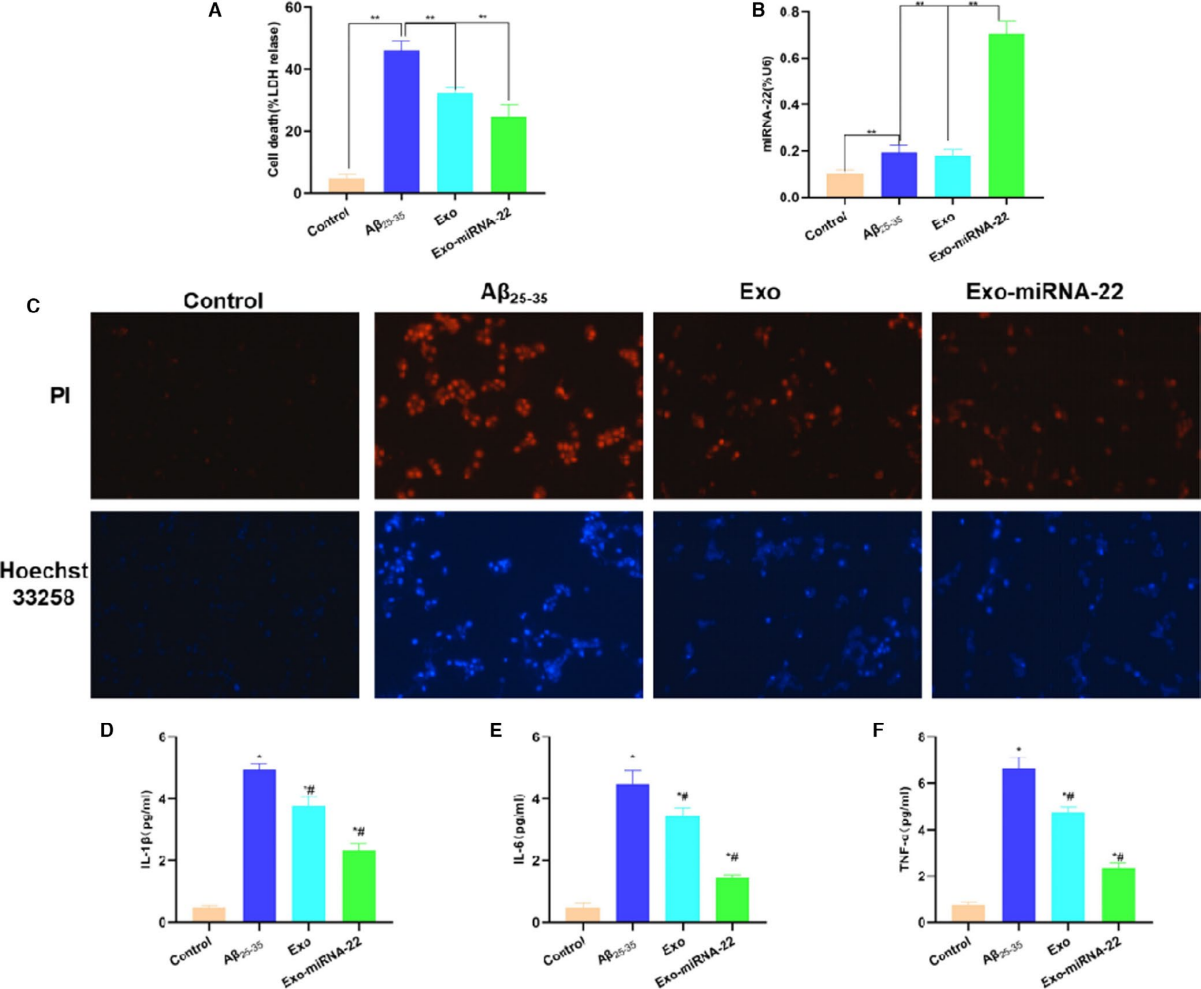
Intervention effect of miRNA‐22‐loaded exosomes on PC12 cell damage. A: Detection of LDH release level ($\bar{x}$±s, n = 3): The LDH release rate was relatively low in the Control group, which was increased in the Aβ_25‐35_ group. Moreover, the LDH release rate was decreased in the Exo and Exo‐miRNA‐22. Comparison between groups, **p <.01. B: Detection of miRNA‐22 levels in cells ($\bar{x}$±s, n = 3): Exo‐miRNA‐22 could significantly increase the level of miRNA‐22. Comparison between groups, **P <.01. C: PI staining and Hoechst 33,258 staining: Cell staining was negative in the Control group. The number of positive cells was significantly increased in the Aβ_25‐35_ group, which was significantly higher than that in the Control group, whereas the number of positive cells was down‐regulated in the Exo and Exo‐miRNA‐22 groups compared with the Aβ_25‐35_ group. D‐F: Detection of inflammatory factors ($\bar{x}$±s, n = 3): Aβ_25‐35_ intervention can promote the release of inflammatory factors. The levels of inflammatory factors were significantly lower in the Exo and Exo‐miRNA‐22 groups than those in the Aβ_25‐35_ group. Comparison with Control group, *p <.05; comparison with the Aβ_25‐35_ group, ^#^ *p* <.05

Aβ_25‐35_ intervention could induce PC12 cell injury in vitro, and the leakage rate of LDH was significantly increased compared with that of the Control group (*p* <.05). The leakage rate of LDH was significantly decreased after Exo and Exo‐miRNA‐22 intervention, which was significantly different from the Aβ_25‐35_ group (*p* <.05). Exo‐miRNA‐22 intervention could further suppress the leakage rate of LDH, which was significantly lower than that of Exo group (*p* <.05) (Figure 4A). The detection of miRNA‐22 showed that Aβ_25‐35_ intervention can increase miRNA‐22 expression, and Exo‐miRNA‐22 intervention could greatly increase miRNA‐22 level, which was significantly different from Aβ_25‐35_ group and Exo group (*p* <.05) (Figure 4B). PI staining and Hoechst 33,258 staining showed negative result in the Control group, suggesting no obvious apoptosis and damage. The number of positive cells was significantly increased in the Aβ_25‐35_ group, and the number of positive cells was significantly decreased in the Exo and Exo‐miRNA‐22 groups, which was significantly different from that in the Aβ_25‐35_ group (*p* <.05), indicating that cell damage was alleviated in the Exo and Exo‐miRNA‐22 groups (Figure 4C). The detection of inflammatory factors showed that the expression of inflammatory factors (IL‐1β, IL‐6 and TNF‐α) was low in the Control group, and Aβ_25‐35_ intervention could significantly increase the level of inflammatory factors, indicating that Aβ_25‐35_ can promote the release of inflammatory factors, whereas the levels of inflammatory factors in the Exo and Exo‐miRNA‐22 groups were significantly decreased, which was significantly different from the Aβ_25‐35_ group (*p* <.05). And the levels of inflammatory factors in the Exo‐miRNA‐22 group were lower than those in the Exo group (Figure 4D–F).

### 3.5 Effects of miRNA‐22‐loaded exosomes on pyroptosis and protein expression in PC cells

**FIGURE 5:**
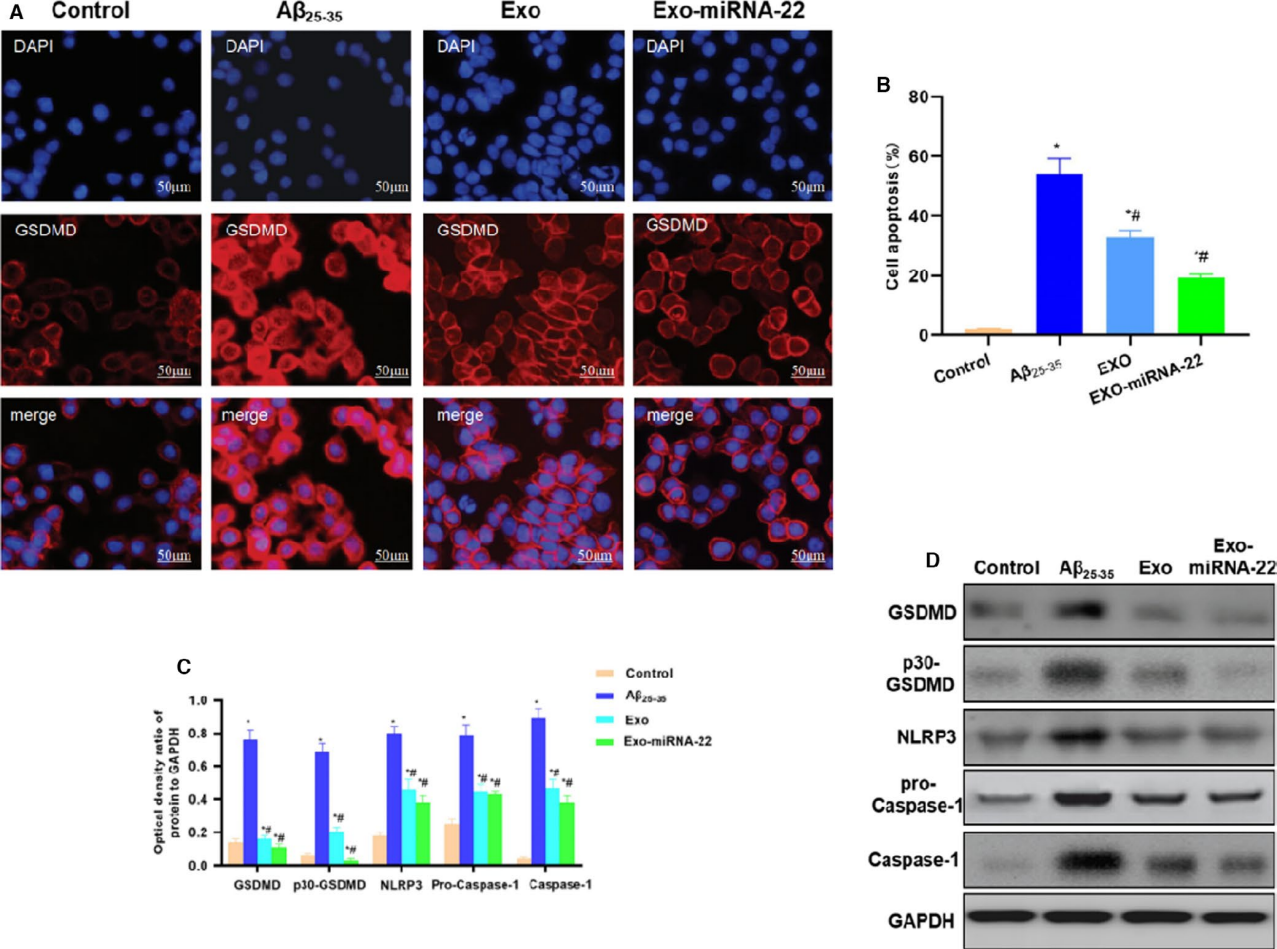
Effects of miRNA‐22‐loaded exosomes on PC12 cell pyroptosis and protein expression. A: Fluorescence staining for GSDMD: The GSDMD expression was low in the Control group, and the fluorescence intensity was low. The GSDMD expression was up‐regulated in the Aβ_25‐35_ group, which was located on the cell membrane and cytoplasm with high fluorescence intensity. The levels of GSDMD in the Exo and Exo‐miRNA‐22 groups were reduced, with decreased fluorescence intensity than in the Aβ_25‐35_ group. C: Flow cytometry results (±s, n = 3): The statistical results showed that the pyroptotic proportion was significantly higher in the Aβ_25‐35_ group than that in the Control group, whereas the pyroptotic proportion was decreased in the Exo and Exo‐miRNA‐22 groups. Comparison with Control group, *p <.05; comparison with Aβ_25‐35_ group, ^#^ *p* <.05. D‐E: Detection of protein expression ($\bar{x}$±s, n = 3): The expression of GSDMD and p30‐GSDMD was low in the Control group, which was significantly up‐regulated in the Aβ_25‐35_ group and significantly down‐regulated in the Exo and Exo‐miRNA‐22 groups. Meanwhile, the expression of NLRP3 inflammasome key protein (NLRP3 and Caspase‐1) was significantly down‐regulated in the Exo and Exo‐miRNA‐22 groups. Comparison with Control group, *p <.05; comparison with Aβ_25‐35_ group, ^#^ *p* <.05

The fluorescence staining of GSDMD protein showed that GSDMD expression level was low in the Control group and was significantly up‐regulated in the Aβ_25‐35_ group, which was expressed in the membrane and cytoplasm, indicating that p30‐GSDMD expression was significantly up‐regulated. However, the expression of GSDM was decreased in the Exo and Exo‐miRNA‐22 group, especially the down‐regulated expression on the membrane, indicating that p30‐GSDMD expression was down‐regulated. However, the fluorescence intensity of GSDMD was lower in the Exo‐miRNA‐22 group than that in the Exo group (Figure 5A). Flow cytometry showed that the pyroptosis rate was lower in the Control group (Annexin V‐FITC+PI+), whereas the pyroptosis rate was significantly increased in the Aβ_25‐35_ group than that of the Control group (*p* <.05), which was significantly down‐regulated in the Exo and Exo‐miRNA‐22 groups, especially in the Exo‐miRNA‐22 group. miRNA‐22 significantly inhibited the expression of GSDMD, and the pyroptosis rate was significantly decreased, compared with the Aβ_25‐35_ group (*p* <.05) (Figure 5B‐C).

Protein detection showed that the expression levels of GSDMD and p30‐GSDMD were lower in the Control group, and the expression of NLRP inflammasome key proteins (NLRP3 and Caspase‐1) was also lower. The expression of GSDMD and p30‐GSDMD was up‐regulated in the Aβ_25‐35_ group, with pyroptosis activation. The expression of NLRP3, Caspase‐1 and pro‐Caspase‐1 in NLRP3 inflammasome was also up‐regulated, which was significantly different from the Control group (*p* <.05). The protein level was significantly down‐regulated in the Exo and Exo‐miRNA‐22 groups, which was significantly different from that in the Control group and Aβ_25‐35_ group (*p* <.05). The levels of GSDMD and p30‐GSDMD were further reduced in the Exo‐miRNA‐22 group, which were lower than those in the Exo group (Figure 5D–E).

## 4 DISCUSSION

AD, a chronic neurodegenerative disease, is clinically manifested as memory disorders, aphasia, impaired visual spatial ability and impaired abstract thinking.^10^ The main pathological changes of AD include the formation of extracellular senile plaques after oligomerization of extracellular amyloid peptide β (Aβ) monomers and neurofibrillary tangles caused by excessive phosphorylation of Tau protein within neurons in brain tissue.^11^ Present studies have also found that the up‐regulation of Aβ protein is closely associated with oxidative stress injury and neuroinflammation. In the cellular inflammatory response, pyroptosis is the research focus in recent years. Pyroptosis is a type of programmed cell death that depends on inflammatory aspartase in host cells.^12^, ^13^ Under harmful stimulation, the signal inside and outside the cell induces the formation of inflammasome in the cytoplasm through the non‐classical Caspase‐1 (Caspase‐1) or Caspase‐4/5/11‐dependent pyroptosis pathway ^14^, ^15^ to activate Caspase‐1 to promote the maturation and release of inflammatory factors (IL‐18, IL‐1β), thereby causing inflammatory response cascade.^16^, ^17^ At present, GSDMD is the most studied in the GSDM family.^18^ Currently, the expression of inflammatory factors, such as IL‐18 and IL‐1β, is found in various cells of in multiple types of nervous systems. Nerve cells can activate Caspase‐1 under different conditions, further mediating pyroptosis.^19^ The role of pyroptosis has been clarified in neurodegenerative diseases, which is manifested in AD, Parkinson's disease and motor neuron disease. The deposition of β‐amyloid protein in AD can cause local inflammation, which can simultaneously cause oxidative stress injury and excitatory injury. Our previous studies have found that β‐amyloid protein can cause pyroptosis in nerve cells by activating Caspase‐1‐GSDMD.^20^ microRNAs are a type of RNAs with 18‐22nt in length. It can inhibit the expression of target genes and protein formation by targeting the 3'‐UTR of mRNA. miRNA‐22 is widely studied. Relevant study has revealed that miRNA‐22 can inhibit the occurrence of pyroptosis and the release of inflammatory factors by regulating GSDMD.^9^

In recent years, stem cell transplantation has been fully studied in AD treatment. It has been found that stem cells (mesenchymal stem cells, MSCs) can inhibit the inflammatory response and cell damage of AD, promote the regeneration of nerve axons and enhance the repair of motor nerves and functional repair. However, due to multi‐differentiation function of stem cells,^21^, ^22^ transplantation may cause side‐effects such as tissue immune rejection, teratogenesis, tumorigenesis; and stem cell survival after transplantation is also a great concern. Therefore, an alternative approach is urgently needed. Exosomes are a type of vesicle‐like structure secreted by cells. Relevant studies have found that MSCs‐derived exosomes also have a good effect on the nerve repair of AD.^23^ To this end, in this study, miRNA‐22 was loaded into ADMSC‐derived exosomes, which was speculated to promote neural cell regeneration and to inhibit neuroinflammation. In APP/PS1 mice (AD mice), both treatment of Exo‐miRNA‐22 and Exo could improve the behavioural and memory abilities of mice, which were significantly different from AD mice. Moreover, the effects of Exo‐miRNA‐22 were more superior. Meanwhile, the expression of inflammatory factors was decreased in peripheral blood and cerebrospinal fluid of mice, and the levels of IL‐6, IL‐1β and TNF‐α in cerebrospinal fluid were especially significantly lower than those of AD mice, which was because miRNA‐22 endocytosis can inhibit GSDMD activation to further improve the release of inflammatory factors. Nissl staining also showed that the denser arrangement of nerve cells in mice of the Exo and Exo‐miRNA‐22 groups than AD mice, and the survival level of nerve cells was also significantly higher than that in AD mice. The above findings definitely indicated that the damage of nerve cells was inhibited, which is closely associated with the improvement of behavioural and memory abilities. The activation of microglia is closely correlated with neuroinflammation. The results also showed that Exo‐miRNA‐22 and Exo could inhibit the activation of microglia, which was manifested by the decreased expression of CD206 and IBA‐1. Of note, Exo‐miRNA‐22 can more significantly inhibit the activation of microglia than Exo, and Exo‐miRNA‐22 can also more significantly suppress the expression of GSDMD and p30‐GSDMD and down‐regulate the expression of NLRP3 and Caspase‐1. Because the cleavage of GSDMD requires the involvement of Caspase‐1, this change is a type of feedback regulation. To further explore the role of Exo‐miRNA‐22, PC12 cells were used in vitro. As a result, Aβ can induce PC12 inflammation and pyroptosis, as well as Caspase‐1‐GSDMD activation. Exo‐miRNA‐22 can inhibit the damage of PC12 cells and the release of inflammatory factors, which were consistent with animal experiments. Therefore, we consider that miRNA‐22‐loaded exosomes can more effectively improve the cognitive ability of AD mice, whose role is associated with nerve repair and inflammatory inhibition. And exosomes are safer than stem cells.

In summary, in this study, we have found that the modification of ADMSC‐derived exosomes by loading miRNA‐22 can improve the cognitive and behavioural capabilities in AD mice, which is associated with the protection of nerve cells and the inhibition of neuroinflammation. Our present finding is a novel therapeutic approach for AD treatment, which deserves further investigation.

## CONFLICT OF INTEREST

All the authors declared no competing interests.

## AUTHOR CONTRIBUTIONS

**Liping Zhai:** Conceptualization (equal); Data curation (equal); Formal analysis (equal). **Heping Shen:** Methodology (equal); Project administration (equal); Resources (equal); Software (equal). **Yongjia Sheng:** Investigation (equal); Methodology (equal); Validation (equal); Visualization (equal). **Qiaobing Guan:** Funding acquisition (equal); Writing‐original draft (equal); Writing‐review & editing (equal).

## Data Availability

The data that support the findings of this study are available from the corresponding author upon reasonable request.
