# Corrigendum

**DOI:** 10.1111/jcmm.17058

**Published:** 2021-12-03

In Liping Zhai et al^1^, the published article contains errors in Figures 3 and 4. The correct figures are shown below. The authors confirm all results and conclusions of this article remain unchanged.

**FIGURE 3:**
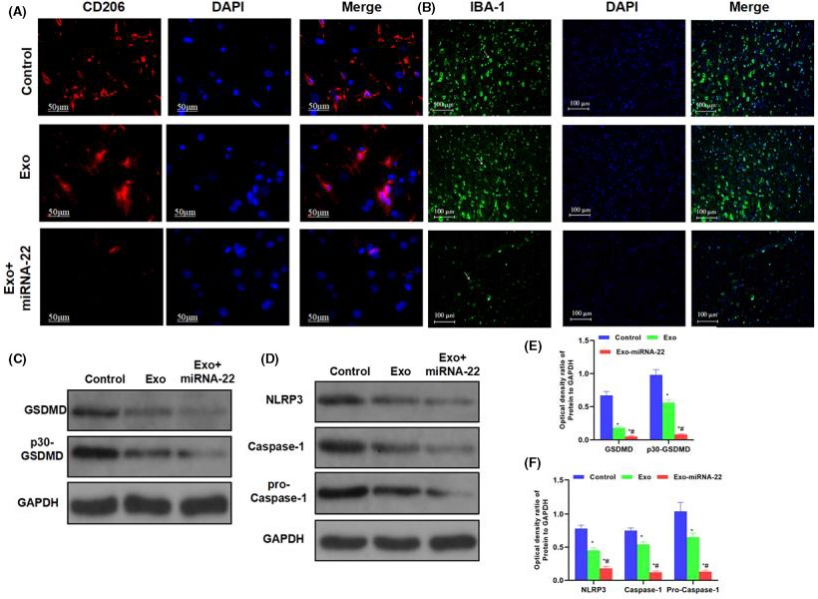
Detection of microglia activation and protein expression in mice. A, B: Immunofluorescence staining of CD206 and IBA‐1 in mice (*n* = 3): The expression of CD206 and IBA‐1 was relatively high in the Control group, with relatively high fluorescence intensity. The expression was downregulated in the Exo group, with decreased fluorescence intensity of CD206 and IBA‐1, which was significantly lower than that in the Control group. The fluorescence intensity of CD206 and IBA‐1 was lower in the Exo‐miRNA‐22 group than that in the Control group and Exo group. C–F, Detection of protein expression ( $\bar{x}$ ± s, *n* = 3): The expression of GSDMD and p30‐GSDMD was relatively high in the Control group, which was significantly downregulated in the Exo and Exo‐miRNA‐22 groups. Meanwhile, the expression of NLRP3 inflammasome key proteins (NLRP3 and Caspase‐1) was relatively high in the Control group, which was significantly downregulated in the Exo and Exo‐miRNA‐22 groups. Comparison with the Control group, *p < 0.05; comparison with the Exo group, #*p* < 0.05

**FIGURE 4:**
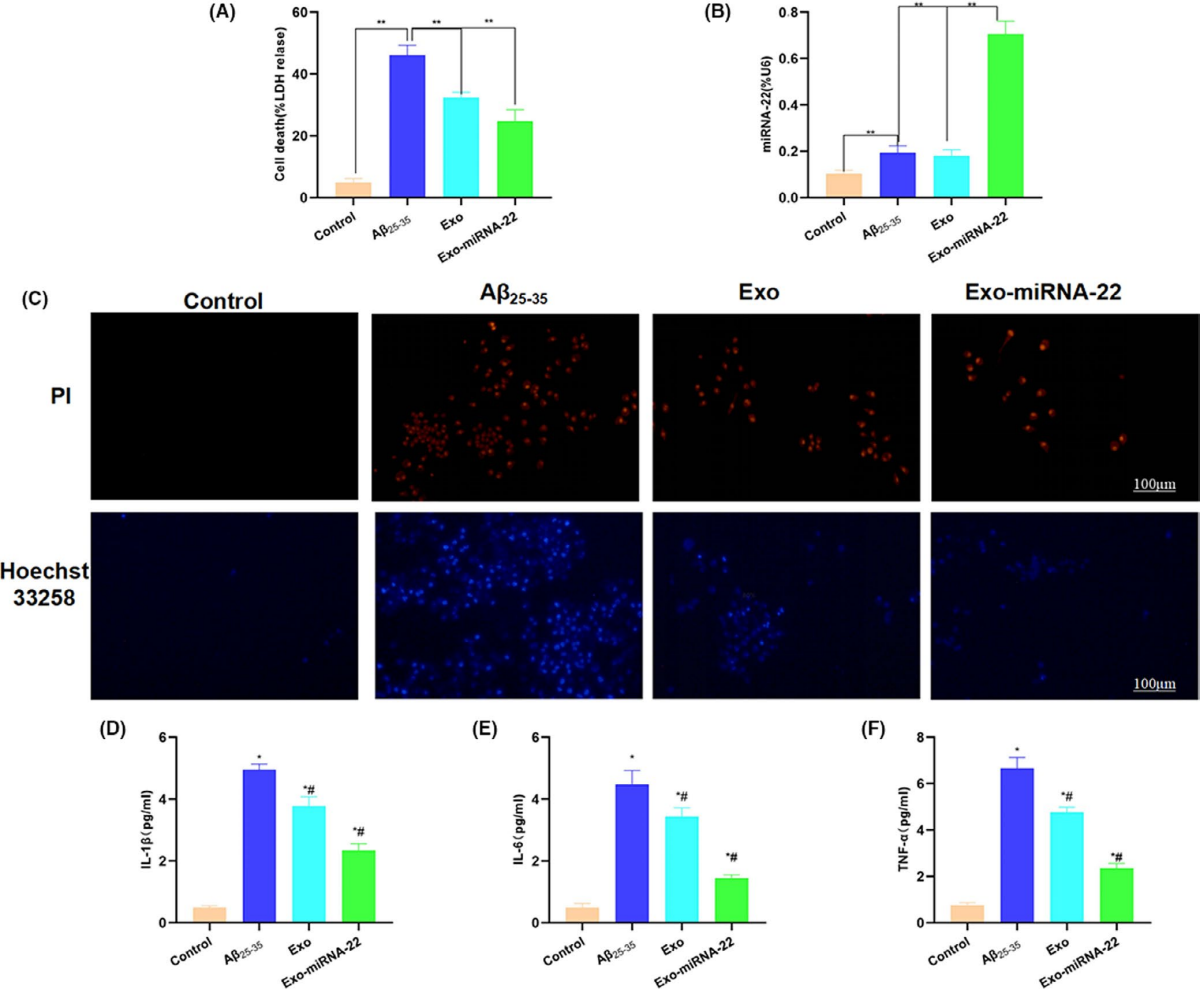
Intervention effect of miRNA‐22‐loaded exosomes on PC12 cell damage. A, Detection of LDH release level ( $\bar{x}$ ± s, *n* = 3): The LDH release rate was relatively low in the Control group, which was increased in the Aβ_25–35_ group. Moreover, the LDH release rate was decreased in the Exo and Exo‐miRNA‐22. Comparison between groups, **p <.01. B, Detection of miRNA‐22 levels in cells ( $\bar{x}$ ± s, *n* = 3): Exo‐miRNA‐22 could significantly increase the level of miRNA‐22. Comparison between groups, **p < 0.01. C, PI staining and Hoechst 33,258 staining: Cell staining was negative in the Control group. The number of positive cells was significantly increased in the Aβ_25–35_ group, which was significantly higher than that in the Control group, whereas the number of positive cells was downregulated in the Exo and Exo‐miRNA‐22 groups compared with the Aβ_25–35_ group. D–F, Detection of inflammatory factors ( $\bar{x}$ ± s, *n* = 3): Aβ_25–35_ intervention can promote the release of inflammatory factors. The levels of inflammatory factors were significantly lower in the Exo and Exo‐miRNA‐22 groups than those in the Aβ_25–35_ group. Comparison with Control group, *p <.05; comparison with the Aβ_25–35_ group, #*p* < 0.05
